# New Understanding of the Difference in Filtration Performance between Anatase and Rutile TiO_2_ Nanoparticles through Blending into Ultrafiltration PSF Membranes

**DOI:** 10.3390/membranes11110841

**Published:** 2021-10-29

**Authors:** Iulian-Gabriel Birsan, Stefan Catalin Pintilie, Laurentia Geanina Pintilie, Andreea Liliana Lazar, Adrian Circiumaru, Stefan Balta

**Affiliations:** 1 Department of Applied Sciences, Cross-Border Faculty, Dunarea de Jos University of Galati, 111th Domneasca Street, 800201 Galati, Romania; iulian.birsan@ugal.ro (I.-G.B.); adrian.circiumaru@ugal.ro (A.C.); 2 Department of Materials and Environmental Engineering, Faculty of Engineering, Dunarea de Jos University of Galati, 111th Domneasca Street, 800201 Galati, Romania or tironglaurentia@yahoo.com (L.G.P.); andreea.lazar@ugal.ro (A.L.L.)

**Keywords:** membrane, polysulfone, TiO_2_, rutile, anatase, ultrafiltration

## Abstract

The blending of nanomaterials into a polymeric matrix is a method known for its ability, under certain circumstances, to lead to an improvement in membrane properties. TiO_2_ nanoparticles have been used in membrane research for the last 20 years and have continuously shown promise in this field of research. Polysulfone (PSf) membranes were obtained through the phase inversion method, with different TiO_2_ nanoparticle concentrations (0, 0.1, 0.5, and 1 wt.%) and two types of TiO_2_ crystalline structure (anatase and rutile), via the addition of commercially available nanopowders. Research showed improvement in all studied properties. In particular, the 0.5 wt.% TiO_2_ rutile membrane recorded an increase in permeability of 139.7% compared to the control membrane. In terms of overall performance, the best nanocomposite membrane demonstrated a performance index increase of 71.1% compared with the control membrane.

## 1. Introduction

Over the years, membrane technology has confirmed its important role in water and gas purification and is still considered one of the best available technologies for these applications. Due to the possibility of adaptation according to a specific destination, membrane separation units could be implemented in industries such as, but not limited to, medical, food, energy, desalination, water treatment, and domestic systems for additional tap water purification and portable filters. However, membrane separation has a major disadvantage, namely fouling caused by contaminants in the feed water, leading to pore blockage and therefore decreasing, generally irreversibly [1], the initial water flux. Among the most extensively studied means of controlling fouling is the incorporation of nanomaterials, metallic or non-metallic nanoparticles, into the membrane structure. In addition to the improved anti-fouling properties this method provided to membranes, it has also been observed to increase water flux and mechanical strength compared to before nanoparticle incorporation. Due to their exceptionally small size, nanomaterials have a high surface-to-volume ratio and high surface energy, which ensures strong adhesion between polymer chains and nanomaterials [2].

Titanium dioxide (TiO_2_) is an interesting semiconductor that has been the focus of many investigations due to its excellent hydrophilicity, photocatalytic properties via absorbed UV light, stability, low cost, and commercial availability [3,4]. Three important crystalline structures of titanium dioxide are known and intensively studied: anatase, rutile, and brookite. The first two forms are considered the most stable [5] and recommended for photocatalysis and disinfection due to their relevant properties and low toxicity to humans [6,7]. The difference between anatase and rutile forms lies in the arrangement of titanium and oxygen atoms in the unit cell. In comparison to the rutile form, the anatase allotrope presents a larger elementary unit, but also a lower density structure [8].

Various scientific articles have reported the fabrication of mixed matrix membranes with TiO_2_ nanoparticles embedded in the membrane matrix, with the results confirming high hydrophilicity, superior flux, and good mechanical strength [9,10]. Despite this, the choice of optimal nanoparticle concentration among other parameters continues to be unclear. Some results are contradictory regarding the exact influence of the manufacturing parameters on the properties of nanocomposite membranes. Some authors detected considerable improvements in the surface hydrophilicity of nanocomposite membranes [11,12], while others observed an insignificant increase [13,14]. In direct opposition to this, hydrophobic tendencies have also been observed with increasing concentrations of TiO_2_ [15,16]. The same contrast was observed with membrane permeability, where the common opinion is the existence of an optimal concentration of TiO_2_ nanoparticles. However, differences among studies are relatively high, from low-optimal concentrations (0–3 wt.%) [15,17,18] to high-optimal concentrations (over 10 wt.%) [19,20]. There are also discrepancies in results regarding the mechanical properties of modified membranes. Various authors have observed optimizations of mechanical properties directly proportional to the concentration of nanoparticles [21]. Yang et al. [11] observed a linear increase in tensile strength with increasing TiO_2_ concentration, from 4.13 MPa (pure membrane) to 6.47 MPa (membrane blended with 5 wt.% TiO_2_). In other studies, it was observed that mechanical strength improved by the addition of nanoparticles up to a certain concentration, followed by reduction in mechanical properties upon more TiO_2_ being added. Yu et al. [22] observed this tendency in tensile strength, with an increase from 1.71 MPa (for a membrane with 18 wt.% PVDF, 5 wt.% PVP, and 0 wt.% TiO_2_) up to 2.16 MPa for a nanocomposite membrane (with 1 wt.% TiO_2_), followed by a decline in mechanical properties at higher nanoparticle concentrations. Thus, an optimal ratio of polymer, solvent, and nanoparticles is required, particularly an optimal concentration of embedded nanoparticles, in order to avoid producing a decrease in mechanical property-related performance. 

Since the first mentions of titanium dioxide nanoparticles in the optimization of membrane properties [13,19,23], numerous studies on their influence have been carried out. However, a study of the literature indicates that the role of the crystalline structure of nanoparticles on the general membrane properties has not been discussed.

The objective of this study is to obtain valuable information on the influence of the crystalline structure of nanoparticles on membrane properties, within the limits of the data provided by these analyses. Two types of titanium dioxide nanoparticles were selected. Both were smaller than 100 nm, with the major difference between these two types of TiO_2_ nanoparticles being their crystalline structure. The first type of nanoparticles had a primary rutile structure, and the second type was a mixture of anatase and rutile, the anatase structure being the majority component, proven by XRD and Raman spectroscopy. Individual tests showed differences between allotropes, although overall performance highlighted a minor contradiction.

## 2. Materials and Methods

### 2.1. Materials Used in Membrane Fabrication

The following materials were used for membrane manufacturing. Polysulfone (PSf, Mw ~ 35,000) was the polymer of choice for the membrane matrix and N-methylpyrrolidone (NMP, C_5_H_9_NO, reagent grade) was chosen as the solvent used to dissolve the polymer. Two different crystalline structured titanium dioxide nanoparticles (particle sizes < 100 nm as validated by the manufacturer), both commercially available, were used as fillers (a mixture of rutile and anatase, and rutile). All the above reagents and materials were purchased from Sigma Aldrich (Darmstadt, Germany). Non-woven support sheets (Novatexx 2471) were kindly supplied by Freudenberg (Weinheim, Germany).

### 2.2. XRD and Raman Analysis for the Studied Nanopowders

The composition of commercial nanopowders was determined with XRD and Raman spectroscopy. Results are provided in the Supplementary Materials.

The X-ray diffraction spectra were compared to standard spectral data via Match! software for phase identification (Crystal Impact, Bonn, Germany). As demonstrated in Figure S1, three phases of TiO_2_ nanoparticles were found using the Crystallography Open Database (COD) correspondence: anatase (COD entry file no.: 96-900-8215), rutile (COD entry file no.: 96-90-4145 and 96-901-5663), and a third matched phase of TiO_2_ (unnamed, COD entry file no.: 96-153-7225). The detected crystalline phase percentages of the first commercial TiO_2_ nanopowder (Figure S1A) were 87.5/12.5 (anatase/rutile), indicating that a high proportion of the powder was of the anatase phase. As expected, the second commercial TiO_2_ nanopowder (Figure S1B) was comprised primarily of the crystalline phase of interest (rutile) with a percentage of 86.3%, with an unnamed TiO_2_ phase comprising 12.5%.

Raman spectra results of the rutile and anatase phases of the titanium dioxide nanoparticles are presented in Figure S2. Both phases, rutile (250.6, 446.3, and 614 cm^−1^) and anatase (404.6, 525.1, 647 cm^−1^), had three peaks with differing Raman vibration fields. As similar values of the two crystalline phases have been reported by other authors [24,25,26], Raman spectroscopy is considered among the most efficient methods of identifying the crystalline structure of titanium dioxide. Additionally, although the composition declared by the manufacturer was a mixture of rutile and anatase, the Raman spectrum of anatase nanoparticles contained characteristic peaks only for the anatase crystalline phase, indicating a satisfactory purity.

### 2.3. Membrane Fabrication

All solutions were homogenized using an electromagnetic stirrer, at room temperature and 1000 rpm, with continuous stirring for 24 h. The nanocomposite membranes were created by incorporating nanomaterials into the solutions of PSf dissolved in NMP. When adding nanomaterials to the solutions, the concentrations of the solvent and polymer were reduced simultaneously to maintain the ratio between the two substances (PSf:NMP = 1:3). The membranes are denoted by the nanoparticle concentration added to the polymer solution, expressed as weight percentage (wt.%), and the predominant crystalline structure for each type of nanoparticle (rutile or anatase), as shown in Table 1.

Before casting, the non-woven substrate layer was fixed to a glass sheet and then wetted with N-methylpyrrolidone. After surplus solvent was removed, the polymer solution was poured onto the wetted substrate. The thin-film polymer solution was then cast using a casting knife (Mitutoyo, Neuss, Germay) with a thickness of 250 µm. Subsequently, the thin film was immersed in a coagulation bath containing distilled water as non-solvent. At this step, phase inversion occurred, where the exchange between solvent and non-solvent resulted in a porous polymeric film, representing the final membrane. After the phase inversion was complete, the membrane was transferred and stored in a container containing distilled water to ensure the solvent residue was completely removed.

In the case of the nanocomposite membranes, the solvent solution was first stirred with the known amount of nanomaterial for one hour, followed by the protocol detailed above for membrane manufacturing.

### 2.4. Characterization Methods

Morphology was investigated using an FEI Quanta 200 (FEI, Hillsboro, OR, USA) scanning electron microscope equipped with an EDX elemental composition analyzer. All samples were coated with gold via sputtering. 

As detailed above, the XRD method was used to determine the crystalline phases of the commercial TiO_2_ nanopowders. Diffraction was obtained with a DRON-3M diffractometer (Saint-Petersburg, Russia), equipped with a molybdenum anticathode (Mo-Kα1). 

A preconfigured Raman spectrometry system (StellarNet, Tampa, FL, USA), composed of a Raman-HR-TEC-785 spectrometer connected to a Ramulaser 785 nm laser, was used to analyze any interactions between the nanomaterial and polymer matrix. It was also used to identify and confirm the molecular structure of polysulfone, along with any possible changes to its structure after the addition of nanoparticles. A total of 10 scans were performed for each type of membrane.

Roughness analysis was performed with an EasyScan 2 AFM (NanoSurf, Liestal, Switzerland) measuring instrument on a 5 × 5 µm area of the surface.

Surface hydrophilicity was evaluated by water contact angle measurement, using a OCA 15EC goniometer (DataPhysics, Filderstadt, Germany). The average value was reported for the water contact angle of each sample. 

Porosity (*ε*) could be determined using a membrane sample with known thickness and surface area. The membrane samples were submerged in water for 24 h, to ensure the sample pores were completely saturated with water, and then weighed to determine the mass of the wet sample. Thereafter, the samples were dried at 40 °C for 24 h to obtain the dry mass. Porosities were determined using the following equation:

(1) $$\varepsilon \;\left(\%\right)=\frac{m_{wet}-m_{dry}}{A\times d\times h},$$

where *m_wet_* and *m_dry_* are the masses of membranes in wet and dry conditions (kg), *A* is the active membrane area (m^2^), *ρ* is the water density (kg m^−3^) and *h* is the membrane thickness (m).

Pure water flux was determined with a HP4750 stirred filtration cell (Sterlitech, Auburn, AL, USA). This parameter was used to measure permeability and membrane compaction degree. Pure water flux (*J_dw_*) was calculated with the following equation:

(2) $$J_{dw}=\frac{V}{A\times t},$$

where *V* is the volume of water that passes through the membrane (m^3^), *A* is the membrane working surface (m^2^) and *t* is filtration time (h). 

Permeability tests were performed with pure water at high operating pressures at room temperature (approx. 25 °C). This parameter was measured after the membranes had undergone compaction produced during the water flux test, to ensure that this phenomenon would not occur during permeability analysis.

Pure water permeability (L m^−2^h^−1^bar^−1^) was calculated using the following expression:

(3) $$P_{dw}=\frac{J_{dw}}{\Delta p},$$

where Δ*p* is the operating pressure (bar).

To perform a linear regression of the measured water fluxes, which defines the membrane permeability, the working pressures were 10, 12, 14, 16, 18, and 20 bar.

The ratio of the initial pure water flux (*J_dw_^initial^*) and the stable pure water flux (*J_dw_^final^*) determined the value of the compaction degree (*CD*), according to the equation:

(4) $$CD=\left(1-\frac{J_{dw}^{final}}{J_{dw}^{initial}}\right)\times 100\;\left(\%\right),$$

Compaction tendency directly influences the permeability of membranes [27].

The separation performance of the studied membranes was analyzed using a feed solution containing 10 ppm Congo Red dye (696.7 g mol^−1^) and distilled water. The concentration of the permeate solution was measured using a DR5000 UV–Vis spectrophotometer (Hach, Loveland, CO, USA). The pressure used in the retention tests of the feed solution was 10 bar.

The retention efficiency (R) of Congo Red dye was calculated using the following equation:

(5) $$R=\left(1-\frac{C}{C_{0}}\right)\times 100,$$

where *C* and *C*_0_ are the dye concentrations in permeate and feed solution (ppm) and R is expressed as a percentage (%).

Fouling resistance of the control and modified membranes are characterized by the relative flux (*RF*) and calculated as follows:

(6) $$RF=\frac{J_{CR}}{Jdw},$$

where *J_CR_* is the retention flux of Congo Red dye solution.

The relative flux reduction (*RFR*) represents the total fouling of membranes, containing both reversible and irreversible fouling types. *RFR* is calculated using the following equation:

(7) $$RFR=\left(1-\frac{J_{CR}}{J_{dw}}\right)\times 100,$$

Analysis of the mechanical properties was performed with a servo-hydraulic testing system Instron 8850 (Instron, Norwood, MA, USA). This enabled the measurement of the elongation-at-break (mm) and tensile strength (MPa). The total dimensions of the tested samples were 2 cm wide and 17 cm long, and the length of the sample subjected to deformation was 10 cm, according to standard ISO 527-1. All tensile tests were performed at a constant traction speed of 5 mm min^−1^ until sample fracture.

For a better comparison between total performances discussed, five properties of interest were selected: permeability, relative flux reduction, retention, tensile strength, and elongation-at-break. The parameters were given as a rating out of 10 for each membrane. For example, membranes with lower permeabilities were given lower ratings, compared to the membrane with the highest permeability. The same process was used for all five parameters of interest. As an observation note, for the relative flux reduction property, the method of converting the value into a rating was inversely proportional, because the highest value meant that the membrane had suffered the highest fouling. Therefore, the membrane with the lowest relative flux reduction obtained the 10 mark. After the conversion of these membrane properties into the form of ratings, these values were displayed in a radar chart to obtain individual performance areas for each membrane. From this chart two area types were extracted: the real areas of performance for membranes, noted as *S_real_*, and an ideal area of performance with all properties marked with 10, noted as *S_ideal_*.

Performance index (*PI*) was calculated using the following equation:

(8) $$PI=\frac{S_{real}}{S_{ideal}},$$

The membrane with the closest performance index to the value 1 is considered the membrane with the best performance. As an additional note, the ideal membrane was based on the limits of the experiments of this study and cannot be related to other studies. Its purpose was to easily and quickly highlight the membrane with the best performance.

## 3. Results and Discussions

### 3.1. SEM and EDX Studies

#### 3.1.1. SEM Surface Observations

**Figure 1:**
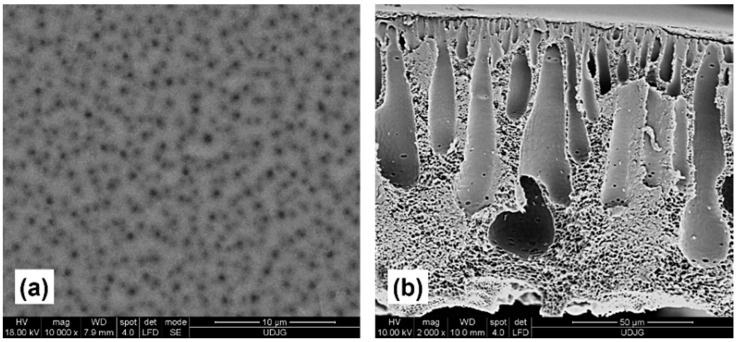
SEM images of control membrane (25 wt.% PSf): (**a**) surface and (**b**) cross-section.

**Figure 2:**
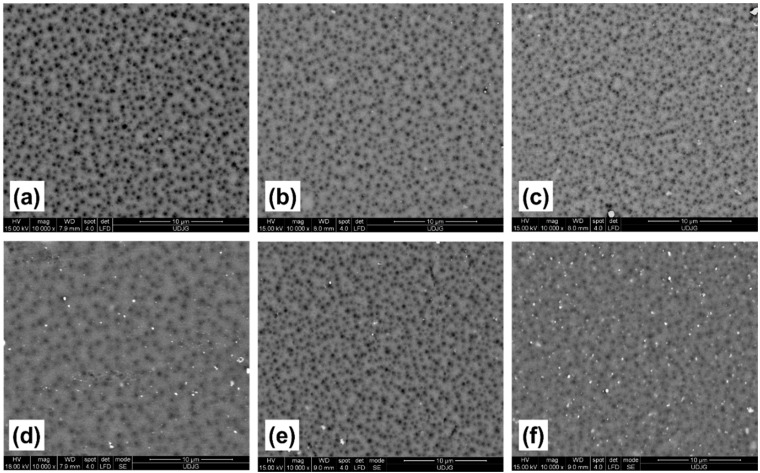
Surface SEM images of nanocomposite membranes modified by the addition of titanium dioxide nanoparticles. Each membrane contains a different concentration, rutile: 0.1 wt.% (**a**), 0.5 wt.% (**b**) and 1 wt.% (**c**); and anatase: 0.1 wt.% (**d**), 0.5 wt.% (**e**) and 1 wt.% (**f**).

In the surface analysis of the control membrane (Figure 1a) and those modified by the addition of 0.1 wt.% TiO_2_ nanoparticles of different crystalline structures (Figure 2a,d), it is observed that the introduction of a small concentration of nanoparticles could greatly influence the surface properties. The number, size, and distribution of surface pores can provide important details regarding water flux and retention percentage. The membrane without added nanoparticles (Figure 1) had both small and large pores, as well as an unfavorable distribution, which can cause low flux and reduced retention.

In the case of nanocomposite membranes with 0.1 wt.% TiO_2_ rutile nanoparticles (Figure 2a), the number of pores increased, while the size distribution remained unchanged, similar to the control membrane. These properties can produce higher flux with retention of contaminants approximately equal to the control membrane.

TiO_2_ anatase nanoparticles influenced the morphology of polymeric membranes differently. In Figure 2d, it can be seen that the nanocomposite membranes modified with 0.1 wt.% TiO_2_ anatase did not have an increased number of pores; instead, the pore size decreased compared to the other membranes. This surface structure can ensure higher retention of the feed solution. Nanoparticles could also be observed on the membrane surface, mainly in the form of agglomerates, which leads to improvement in the hydrophilic character due to the high hydrophilicity of nanoparticles. 

In the case of nanocomposite membranes with a concentration of 0.5 wt.% TiO_2_ rutile and anatase nanoparticles (Figure 2b,e), it can be observed that the number of pores increased, while their average size decreased. These changes in the morphology of the membranes can increase the water flux, but also result in higher retention of the feed solution.

SEM micrographs of the upper layer of the membrane obtained by the incorporation of 1 wt.% TiO_2_ nanoparticles in the polymeric matrix, are shown in Figure 2c,f. An increase in the density of nanoparticle agglomerates, directly proportional to the mass percentage added into the membrane matrix, is observed. Agglomerations were more pronounced in the case of membranes with embedded nanoparticles of anatase crystalline structure. Nanocomposite membranes with 1 wt.% TiO_2_ rutile had smaller pores and a pore density greater than the membranes with 1 wt.% TiO_2_ anatase. The pore size was also observed to decrease in proportion to the percentage of nanoparticles initially added to the polymer solution, regardless of the crystalline structure. Similar trends have been reported by other authors [28,29].

The tendency of nanoparticles to agglomerate on the surface of the studied membranes was greater in the case of membranes with the addition of TiO_2_ anatase nanoparticles. 

Pores were also observed in the vicinity of nanoparticles or groups of nanoparticles, which could mean that nanoparticles enhance pore forming during the process of membrane formation. This may explain their higher number compared to the pure membrane. Thus, a higher concentration of nanoparticles led to higher pore density.

#### 3.1.2. Cross-Section Observations

**Figure 3:**
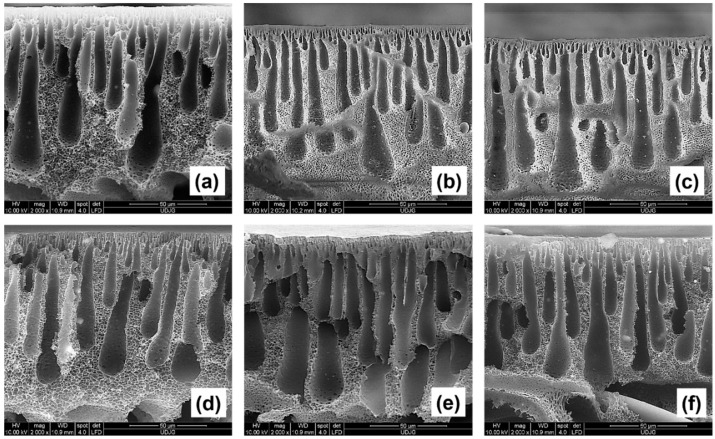
Cross-section SEM images of nanocomposite membranes modified by the addition of titanium dioxide nanoparticles. Each membrane contains a different concentration, rutile: 0.1 wt.% (**a**), 0.5 wt.% (**b**) and 1 wt.% (**c**); and anatase: 0.1 wt.% (**d**), 0.5 wt.% (**e**) and 1 wt.% (**f**).

Cross-section SEM images of membranes with concentrations of 0.1 wt.% TiO_2_ rutile and anatase nanoparticles are illustrated in Figure 3a,d. The membrane morphologies indicate that their modification did not produce noticeable differences compared to the control membrane (Figure 1b).

All membranes had elongated macrovoids that were larger at the base than the top, predominantly drop-shaped, in which a smooth connection was established between the upper and lower layers of the membrane. Moreover, the thickness of the active layer did not show observable changes.

Although changes in surface morphology were observed in the presence of 0.1 wt.% TiO_2_ nanoparticles (Figure 2a,d), the exchange between solvent and non-solvent during phase inversion was not sufficiently influenced by the presence of nanoparticles for the polymeric solution to be able to modify the internal membrane structures.

For those membranes incorporating 0.5 wt.% nanoparticles (Figure 3b,e), macrovoid length increased, with a narrower base and a finger-like structure. The active upper layer was thinner and denser, which can lead to better permeation properties. According to Shukla et al. [30], membrane morphology is dependent on the percentage of nanomaterial added. This is due to the increased hydrophilicity resulting from different concentrations of nanomaterial, where increasing nanoparticle concentration leads to an increased exchange rate between solvent and non-solvent during immersion precipitation, thus resulting in a membrane with high porosity and more developed macrovoids.

As can be seen in Figure 3c,f, the cross-section images of the modified membranes with 1 wt.% nanoparticles show sections of elongated finger-like pores, with greater uniformity compared to membranes containing lower nanoparticle concentrations. In addition, the upper layer became denser and less thick compared to the control membrane.

#### 3.1.3. EDX Observations

The results of the surface EDX elemental analysis of titanium for membranes with added rutile and anatase nanoparticles of different concentrations are presented in Table 2a. The data presented in Table 2 reveal an interesting fact. The detected titanium concentrations on the surfaces of the two membrane types modified with 0.1 wt.% titanium dioxide were very different. In the case of rutile, the detected titanium concentration was approximately three times lower than in the case of anatase. This means that the rutile-modified membrane did not contain, at least on the surface, the expected amount of titanium dioxide. Corroborating this observation with the crystalline structure of the two allotropic forms of titanium dioxide and the data presented regarding the two crystalline structures, it is possible that during phase inversion much of the amount of rutile nanopowder was washed and entrained simultaneously with the water, a phenomenon called leaching.

**Figure 4:**
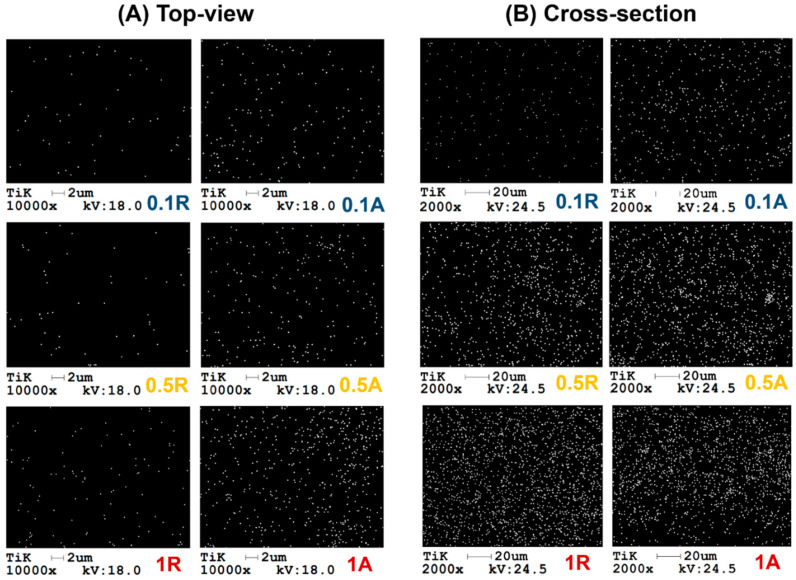
Surface (**A**) and cross-section (**B**) EDX maps of the Ti element for nanocomposite membranes.

As can be observed visually from the SEM images (Figure 2) and EDX surface maps of the titanium element (Figure 4A), the table values confirm that the membrane with TiO_2_ anatase nanoparticle addition presented a higher percentage of nanoparticles on the surface.

The cause for these differences in surface concentration can also be explained by the differences in hydrophilicity between the rutile and anatase nanoparticles, trends proven experimentally by other authors from distinct scientific fields. According to the study of Yin and colleagues [24] on the role of crystalline structures (anatase, rutile, and brookite) on TiO_2_ films, deposited on a borosilicate glass layer used in photocatalysis, they observed that the thin film composed of rutile presented a higher water contact angle than the anatase composite film, under both normal and UV illumination environments. This indicates that the anatase crystalline structure is more hydrophilic than that of rutile. In another study, Bolis et al. [31] analyzed the hydrophilic/hydrophobic properties of commercial TiO_2_ nanoparticles for use as inorganic additives in sun protection lotions. The hydrophilic nature of the dry powders was dependent on the crystalline phase, the highest affinity for water being reported in the anatase structure. It can be considered that the anatase nanoparticles used in the present study have higher hydrophilicity than the rutile nanoparticles. Therefore, the excessive presence of anatase nanoparticles on the upper layer of membranes originates from the moment of initiation of phase inversion of the thin films from the solvent–polymer–nanoparticle system. When immersing the thin film in the coagulation bath (composed entirely of distilled water), the exchange between solvent (n-methylpyrrolidone) and non-solvent (distilled water) occurred at the point where the solvent dissolved in the coagulation bath. Simultaneously, due to the superior hydrophilic nature of anatase nanoparticles, they migrated from their initial position towards the water-rich area. More precisely, that area is at the polymer–water interface where the upper layer of the membrane is formed. The phenomenon of migration of nanoparticles to the upper layer of the membrane has been debated in other works [32,33].

In the subsequent cross-section elemental analysis of membranes modified with TiO_2_ nanoparticles of different concentrations and crystalline structures (Table 2b), it is observed that there was an increased percentage of nanoparticles detected by EDX analysis, compared to the initial quantity added in the polymeric solutions, thus indicating their agglomeration behavior.

From the correlation of elemental percentages detected using the surface EDX method (Table 2a) with those of the membrane cross-section (Table 2b), it is observed that the presence of anatase TiO_2_ nanoparticles on the membrane surfaces was relatively close in the case of low additive content membranes and moderately higher in the case of high additive content membranes when compared to the mass percentage found in the cross-sections. These discrepancies between membrane surface and cross-section confirm the migration of nanoparticles to the membrane surface. 

As in the case of the surface analyses, the data in Table 2b show that the presence of titanium in the modified membrane with 0.1 wt.% rutile was five times smaller than that of titanium in the membrane with 0.1 wt.% anatase. For the other two concentrations (0.5 wt.% and 1 wt.%) it is observed that the presence of the titanium was comparable.

To be noted is the fact that, due to the higher leaching tendency of the rutile nanoparticles during phase inversion, the EDX map results could signify that the chemical bonding between rutile titanium and the polymer matrix was weaker than in the case of the anatase–polymer system, due to differences in hydrophilicity between the two allotropes. This is partially proven by the Raman analysis of the studied membranes (see Section 3.2).

Because of the relatively high percentage of titanium present in 1 wt.% TiO_2_ anatase and rutile blended membranes, the nanoparticles would likely cause blockages in the membrane’s internal pores, resulting in a lower water flux than the 0.5 wt.% nanoparticle modified membranes. This phenomenon has been discussed by other authors [34,35].

According to the surface EDX maps corresponding to the titanium element, shown in Figure 4A, it is observed that the distribution of nanoparticles was uniform. The presence of titanium corresponding to nanoparticles with anatase crystalline phase was higher than in the membrane with added rutile TiO_2_. 

The presence of nanoparticles on the membrane surface can influence the hydrophilic character but also the roughness. A high concentration can increase the affinity for water, which is desired in membrane properties, but at the same time, it can result in a rougher surface. This can cause an increase in fouling during filtration processes [36].

In Figure 4B the distribution of the Ti element in the membrane structure is shown. An important aspect observed in most of the maps extracted from the EDX analysis is that the distribution of titanium dioxide nanoparticles was uniform throughout the membrane section area. It is visible that, in the investigated area, the distribution of dots corresponding to titanium was less dense in the case of 0.1 wt.% rutile than in the case of 0.1 wt.% anatase. For the other two distributions, comparable densities could be observed, corroborating the data presented in Table 2b.

In general, the presence of nanoparticles in a uniform regime throughout the section of nanocomposite materials is a governing factor in successfully optimizing the desired properties, such as mechanical properties [37].

However, it can be observed in the case of the nanocomposite membrane with 1 wt.% TiO_2_ anatase that, at the bottom of the cross-section, the nanoparticle density was lower than in the upper area. This phenomenon is in line with the higher frequency of anatase nanoparticles on the surface of 1 wt.% TiO_2_ membrane, where the migration of the nanoparticles was evident, compared to the 1 wt.% rutile TiO_2_ nanoparticle blended membrane. This type of migration was also observed in EDX analysis by Hosseini et al. [38], which investigated the influence of titanium dioxide nanoparticles at higher concentrations (5–10 wt.%) on the properties of membranes composed of polyethersulfone at lower concentrations (13, 15 and 17 wt.% PES).

### 3.2. Raman Spectroscopy of Membranes

**Figure 5:**
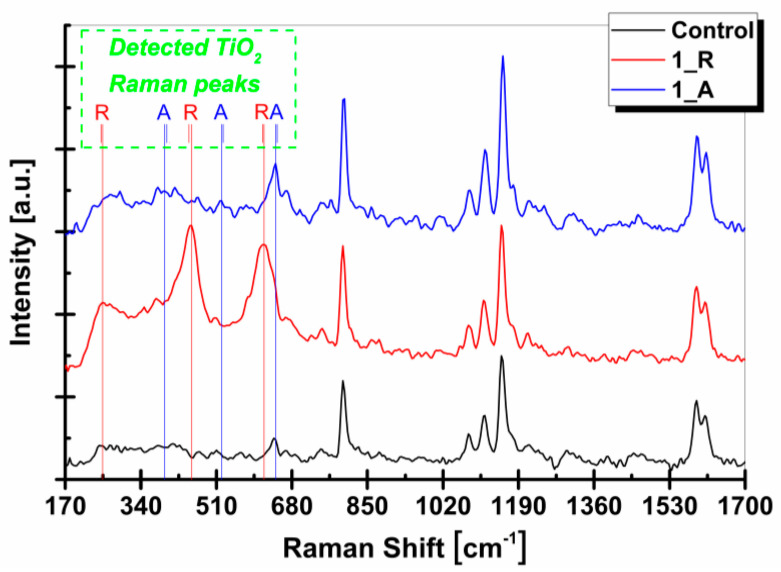
Raman spectra of membranes with 1 wt.% TiO_2_ rutile and anatase nanoparticles and control membrane. Note: the vertical lines that intersect the graphic represent the detected wavenumbers of anatase and rutile peaks in the nanocomposite membrane; the short vertical lines represent the detected wavenumbers of anatase and rutile peaks in the nanopowders.

The possible nanoparticle–matrix interactions at the molecular level can be highlighted by comparing the Raman spectra of the control membrane with the nanocomposite membranes with the addition of 1 wt.% TiO_2_ nanoparticles, as seen in Figure 5.

The Raman wavenumbers of the polysulfone spectral fingerprint can be identified, namely 644, 795, 1080, 1114, 1151, and 1591 cm^−1^, similar to peaks observed by other authors [39,40,41]. Table 3 shows the correlations of the Raman wave domains with the possible functional groups related to the polymer in this study.

The wavenumber range of 700–1800 cm^−1^ did not show differences between the spectra corresponding to the composite membranes and the membrane without nanoparticles, with only values characteristic to polysulfone being observed. Changes in Raman spectra produced by the presence of titanium dioxide nanoparticles occurred in the range 200–700 cm^−1^. Relevant is the fact that Raman analyzes provided clear evidence that the incorporation of TiO_2_ nanoparticles did not alter the chemical structure of the polysulfone membrane.

In the Raman spectrum of modified membranes by the addition of rutile TiO_2_ nanoparticles, additional bands appeared at 251 cm^−1^, 446 cm^−1^, and 614 cm^−1^. These vibration bands are similar to the Raman spectrum of the rutile TiO_2_ nanoparticles, which denotes that the rutile’s chemical structure had not been changed. 

In the case of 1 wt.% TiO_2_ anatase membranes, it can be observed that the Raman band intensities specific to the fingerprint of anatase were not as high as those of the rutile TiO_2_ nanoparticles. All three Raman bands were found in the nanocomposite membranes but at reduced intensities, indicating a certain contribution of anatase TiO_2_ nanoparticles to the polymer matrix. It is reasonably possible that one of the vibration modes of TiO_2_ in the anatase structure was blocked by a chemical bond with the polymer, namely the one characterized by 525.1 cm^−1^.

It can be observed that two peaks were different from those that are characteristic of titanium dioxide (anatase allotropic form), so that the disappearance of the aforementioned peak together with the peak attenuation of 412 cm^−1^ specific to the anatase form could be more confidently attributed to the blocking of the two vibration modes, blocking which cannot be the result of a superficial (interfacial) physical interaction. The third peak of the anatase structure (647 cm^−1^) was in the approximate vicinity of one characteristic peak of the polymer matrix (644 cm^−1^), creating an overlap in Raman peaks, the only distinction between nanocomposite membrane and control membrane being the intensity increase due to TiO_2_ anatase nanoparticle addition [39].

Following Raman results, the band intensities associated with rutile nanoparticles were more pronounced in the spectral band of nanocomposite membranes, compared to those of the anatase form. Because of this aspect, but also due to the more efficient distribution of the rutile nanoparticles on the surface and in the membrane sections proved by the absence of visible excessive nanoparticle agglomerations, it can be noted that the influence of the rutile crystalline phase on the polysulfone, and therefore the membranes used in this study, could be more favorable than that of anatase nanoparticles.

Correlating the Raman intensities resulting from the presence of nanoparticles in the polymeric matrix with those determined by the EDX method (Table 2a), TiO_2_ rutile nanoparticles created better-defined links from a physical point of view, while TiO_2_ anatase nanoparticles led particularly to the creation of chemical bonds of a nanoparticle–matrix type. These aspects can cause differences in the mechanical behavior of nanocomposite membranes. If the nanomaterial is not fixed, it will behave as a set of defects randomly distributed in the polymeric matrix, therefore contributing to an uneven distribution of stress along the polymeric chains with consequences in altering the macroscopic mechanical response.

### 3.3. Hydrophilicity, Porosity, and Roughness

#### 3.3.1. Water Contact Angle

The water contact angle is a method for determining the hydrophilic character of membranes. As can be identified in Table 4, the highest contact angle value was obtained by the control membrane. Because of the high hydrophilicity characteristic of nanoparticles, when they are present in the membrane matrix, regardless of the mass percentage chosen, the contact angle decreases, which in response will improve the membrane’s hydrophilic character.

The membranes with the addition of TiO_2_ anatase had lower water contact angle values. An explanation is provided by the surface SEM and EDX data, where the presence of anatase nanoparticles was higher compared to membranes modified with TiO_2_ rutile, which can lead to a higher affinity for water.

When comparing only the nanocomposite membranes, it can be observed that the highest water contact angle corresponded to the membrane with 0.1 wt.% rutile, which was known to have reduced nanoparticle content. The difference in measurement, compared to the control membrane, was 7° which means that the effect of titanium dioxide nanoparticles (rutile phase) was less significant, as expected since their density was considerably lower than that of anatase titanium dioxide nanoparticles.

The lowest water contact angle values were achieved by the membranes blended with the highest nanoparticle concentration, namely 1 wt.% TiO_2_ anatase and 1 wt.% TiO_2_ rutile.

#### 3.3.2. Porosity

Porosity represents a method of measuring the total volume of membrane macrovoids, in which the polymer is not present. Put differently, this method offers an objective value for structural analyses of membrane surfaces and cross-sections obtained using SEM microscopy.

As can be seen in Table 4, from the crystalline structure point of view, the membrane porosities did not differ significantly. This fact was also observed in the cross-section SEM images. However, because the membranes blended with rutile TiO_2_ had slightly higher porosity values, they had a higher water flux than the membranes with the addition of anatase-phase nanoparticles.

Regarding nanoparticle concentrations, increases in the quantity of TiO_2_ nanoparticles contributed to porosity increases. It can be observed that the incorporation of 0.1 wt.% TiO_2_ (rutile or anatase) into the nanocomposite membranes produced a porosity approximately equal to that of the control membrane, values that corroborate the cross-section SEM images (Figure 2 and Figure 3), where changes in structure were not significant.

The leaching hypothesis of titanium dioxide’s rutile allotropic form during the phase inversion is also confirmed by the percentage values of the modified membrane porosities (Table 4), where it can be seen that the porosity of the membranes modified with rutile TiO_2_ was higher than in the case of the anatase form, contributing not only to the formation of surface pores but also to the lengthening of microvoids in the membrane structure.

Yang and co-workers [45] stated that an adequate addition of TiO_2_ nanoparticles could improve porosity and increase the number of pores, achieving a significant increase in water flux and an unchanged degree of retention.

#### 3.3.3. AFM Observations

**Figure 6:**
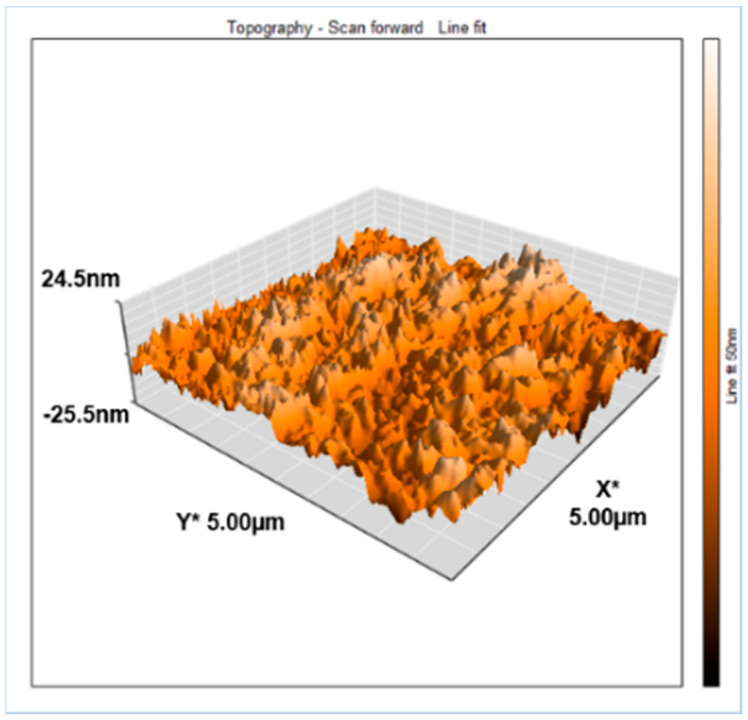
3D AFM image of the control membrane.

Determining the membrane roughness and topography is necessary, as this provides significant details in understanding the membrane fouling behavior during filtration [13,46]. In the case of the control membrane (Figure 6), the deep valleys represent the porous structure, while the peaks are identified as nodules of the membrane surface [47,48].

**Figure 7:**
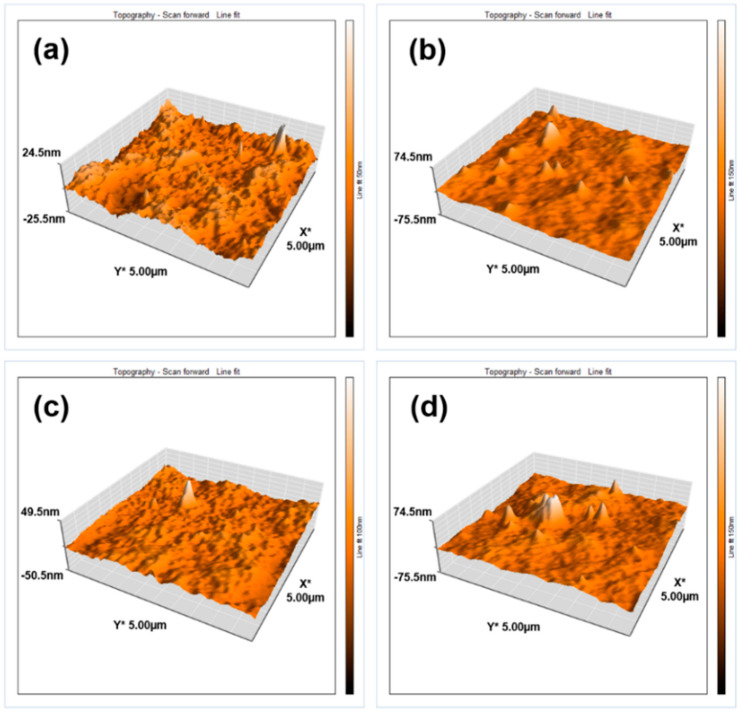
AFM 3D roughness for membranes with: (**a**) 0.1 wt.% TiO_2_ Rutile, (**b**) 1 wt.% TiO_2_ Rutile, (**c**) 0.1 wt.% TiO_2_ Anatase, (**d**) 1 wt.% TiO_2_ Anatase.

Figure 7 shows three-dimensional images of the membranes with the addition of TiO_2_ anatase and rutile nanoparticles at different concentrations. The surface of the control membrane had a uniform topography of higher apparent roughness than the membrane with 0.1 wt.% TiO_2_ rutile (Figure 7a). 

In order to easily observe surface topography, the Z-scale was set for every sample individually. It can be seen that the highest Z-values were for the highest concentration of nanoparticles. Setting all topographies to the same Z-scale would have led to the attenuation of many peaks or valleys, which was not the interest of this study.

Pronounced peaks can be observed, as well as a smoother surface resulting from the incorporation of 0.1 wt.% TiO_2_ nanoparticles, both anatase and rutile (Figure 7a,c). The explanation provided by Yogarathinam et al. [47] regarding this surface smoothing attributes it to the addition of nanoparticles into the polymeric solution, which leads to the regularization of the transfer between solvent and non-solvent during phase inversion, proceeding to the formation of a uniform porous structure at the surface. The membranes modified by incorporation of 1 wt.% TiO_2_ anatase (Figure 7d) provided a high surface area associated with formations of nanoparticle agglomerations, with a rougher surface compared to the membranes blended with 1 wt.% rutile nanoparticles (Figure 7b); this corroborates a phenomenon observed in other works, where the agglomeration tendency increases concomitantly with the concentration of added nanoparticles [16].

The average roughness (Sa) values of the studied membrane are shown in Table 4. A reason for the increase in membrane roughnesses with the addition of 1 wt.% TiO_2_ rutile and anatase may be the increase in the number of surface pores observed in the surface SEM images of Figure 2c,f as well as the higher total porosity values (Table 4), which may lead to decreasing fouling resistance. Similar results regarding roughness tendency and its correlation with decrease in fouling resistance have been reported by other authors [20,49].

### 3.4. Permeation Properties

#### 3.4.1. Permeability

**Figure 8:**
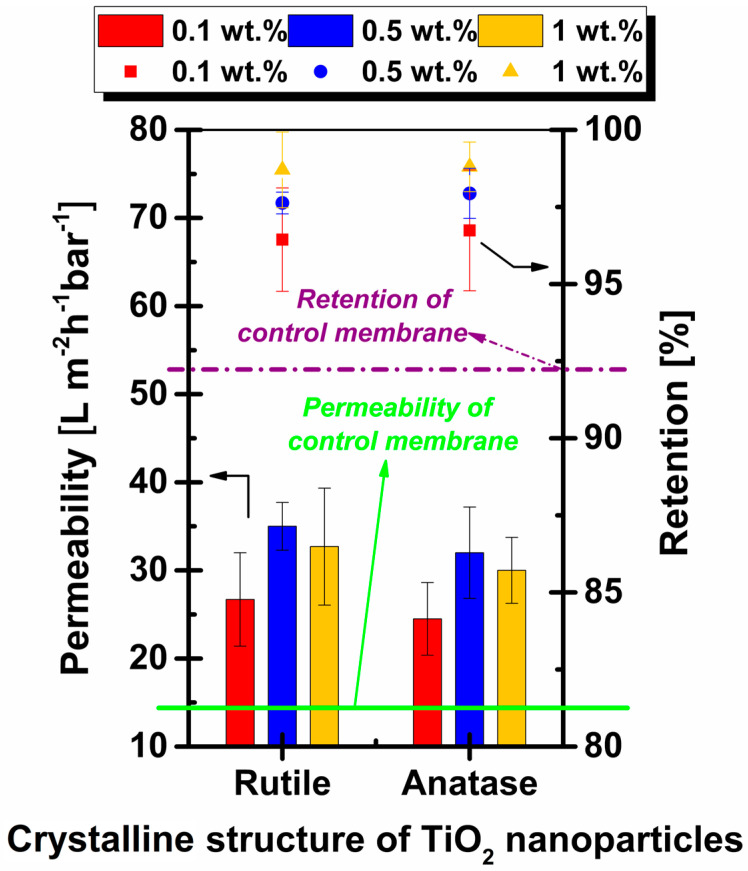
Permeability and retention of the control membrane and membranes with the addition of rutile and anatase nanoparticles at concentrations of 0.1 wt.%, 0.5 wt.% and 1 wt.%.

The addition of a very small mass percentage (0.1 wt.%) of titanium dioxide nanoparticles led to a substantial increase in permeability. In Figure 8 it is observed that the permeability of 0.1 wt.% TiO_2_ rutile membranes increased approximately twofold compared to the permeability of the control membrane. These outcomes are in accordance with the surface SEM micrographs, where the addition of 0.1 wt.% TiO_2_ rutile enhanced the number of pores during phase inversion. These changes achieved an increase in permeability even at distinctly low concentrations, a phenomenon observed by other authors [16,50]. 

The most significant difference, taking into account both crystalline structure and nanoparticle concentration, occurred between the membranes with 0.5 wt.% TiO_2_. Rutile blended membranes achieved a permeability 9.4% higher than membranes modified with anatase nanoparticles. However, compared to the control membrane, the incorporation of 0.5 wt.% TiO_2_ rutile and anatase nanoparticles into the polymeric solution led to increases in permeability of 139.7% and 119.2% respectively.

Increased porosity, as well as pore density on the surface of nanocomposite membranes, resulted in the optimization of permeation properties [51].

The permeability values recorded for the membranes with the addition of TiO_2_ rutile were higher, regardless of concentration, compared to TiO_2_ anatase membranes. As discussed earlier, one role of nanoparticles roles is pore forming, but at the same time at higher concentrations, they tend to develop agglomerations which can lead to decreased membrane performance. The mass percentage of 1 wt.% TiO_2_ in the polymeric solution led to a decrease in permeability. The most accepted explanation provided by literature on the decrease in permeability at higher nanoparticle concentration is the blocking of membrane pores with nanoparticles or nanoparticle agglomerations [52,53].

#### 3.4.2. Retention Tests

In the membrane filtration tests, a solution containing 10 ppm concentration of Congo Red dye was used.

Figure 8 shows that retention of Congo Red increased concomitantly with the percentage of nanoparticles present in the membrane. The highest retentions were achieved by 1 wt.% TiO_2_ blended membranes, with values of 98.71% and 98.80% for the rutile and anatase nanoparticles, respectively. As the pore size decreased, the retention rate of the membranes increased. This tendency has also been observed by other researchers in this field [12,15]. The decrease in pore size with the increase in nanoparticle concentration was observed in the surface image analysis provided by SEM (Figure 1 and Figure 2), which may be correlated with the retention results.

Although the addition of 1 wt.% nanoparticles led to a decrease in permeability due to the pore blockage tendency of nanoparticles, there was no decrease in separation performance in the retention tests of the Congo Red solution. Good retention means ensuring high-quality water produced through a membrane process.

#### 3.4.3. Relative Flux

**Figure 9:**
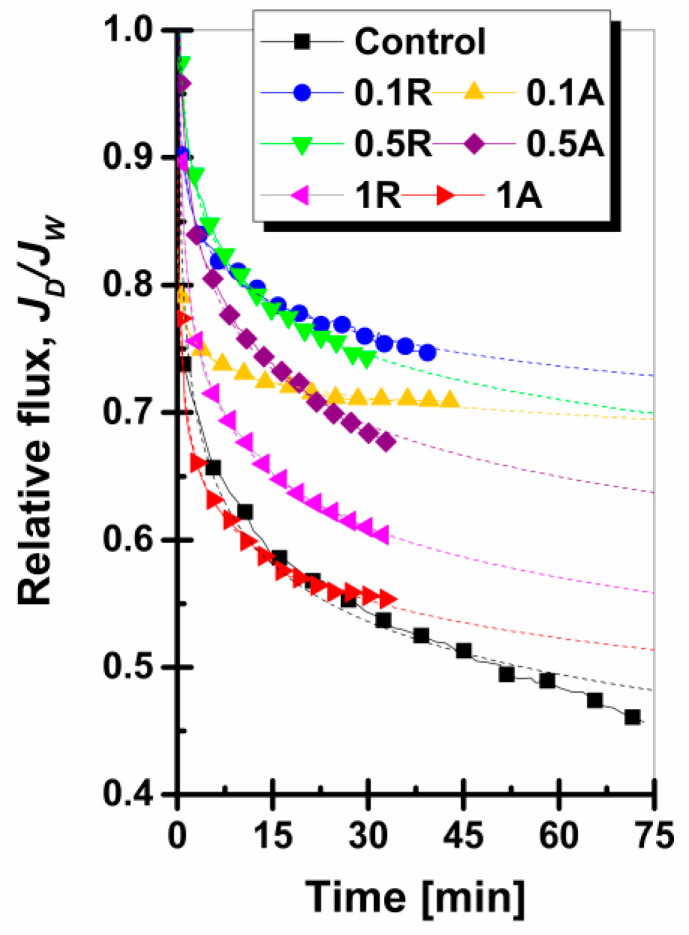
Study of membrane fouling using the relative flux method for the control membrane and the membranes with the addition of TiO_2_ nanoparticles, rutile and anatase phases (Note: dashed lines represent the allometric non-linear curve fittings of the relative fluxes).

The relative flux of membranes during filtration is a method of studying the membrane fouling behavior. Fouling causes substantial flux loss, which requires increased working pressure to ensure a constant flux, as well as additional chemical treatments and regular shutdowns for membrane cleaning [54]. The membrane’s ability to withstand fouling is dependent on surface chemistry, roughness, and hydrophilic character [55]. A high relative flux of membranes indicates better fouling resistance abilities [56]. The relative fluxes corresponding to the nanocomposite membranes are shown in Figure 9. By analyzing the fouling evolution during the entire filtration process, certain observations can be adopted.

The relative flux of nanocomposite membranes increased for nanoparticle concentrations of 0.1 wt.% and 0.5 wt.% and then decreased noticeably for concentrations of 1 wt.% TiO_2_ in both rutile and anatase forms. 

The relative flux of the control membrane showed the greatest decrease over time. Celik et al. [57] explained this phenomenon as being caused by the increased hydrophobic interactions between the membrane and the contaminant. This contributes to the rapid deposition of dye molecules on the membrane surface as well as in its pores. 

The inclusion of a minimal amount of nanoparticles, 0.1 wt.% TiO_2_, resulted in the optimization of the membrane surface. This increase in fouling resistance was closely connected with the low roughness as well as the enhancement of the hydrophilic character [58]. In the comparative analysis between the rutile and anatase crystalline structures of the 0.1 wt.% TiO_2_ membranes, it was observed that the rutile TiO_2_ membrane produced a stronger relative flux. In contrast, the 0.1 wt.% TiO_2_ anatase-modified membrane provided better relative flux stability over time.

The higher relative flux values of rutile TiO_2_ membranes were governed by the lower roughness. The superior relative flux stability over time of the anatase TiO_2_ membranes was the result of the lower water contact angles. Membranes with the addition of 0.5 wt.% TiO_2_ indicated similar relative flux values as the lower concentration of nanoparticles, but the stability of the relative flux over time was lower. This aspect may be caused by the relatively higher roughness values than the membranes with 0.1 wt.% TiO_2_. In the case of membranes with the addition of 1 wt.% TiO_2_ nanoparticles, the relative flux decline was the most pronounced, mainly due to the increase in roughness.

#### 3.4.4. Relative Flux Reduction (RFR)

Relative flux reduction is a practical tool in identifying the membrane with the best resistance to fouling. According to Table 5, as expected, the control membrane showed the highest degree of fouling with a value of 54.3%. Blending nanoparticles into the polymer solution led to the creation of membranes with better anti-fouling properties. The lowest RFR value was realized by the membranes with 0.1 wt% TiO_2_ rutile, with a value of 24.9%. 

It can be observed that the membranes with nanoparticle concentrations of 0.1 wt.% and 0.5 wt.% showed similar results. The increased RFR value for the high nanoparticle concentration membranes (1 wt.% TiO_2_ NPs) indicates that these membranes suffered a high fouling degree. At this concentration, the influence of membrane surface roughness was more important in fouling control than the membrane’s hydrophilic property.

### 3.5. Mechanical Properties

An appropriate concentration of titanium dioxide nanoparticles can optimize mechanical properties, such as tensile strength and elongation-at-break. These efficiencies are necessary for the prevention of possible defects caused by high pressures during water filtration in engineering applications.

The casting of the polymeric solution in thin films on the support layer increased the tensile strength of the membranes but produced a decrease in elongation-at-break.

#### 3.5.1. Elongation-at-Break

**Figure 10:**
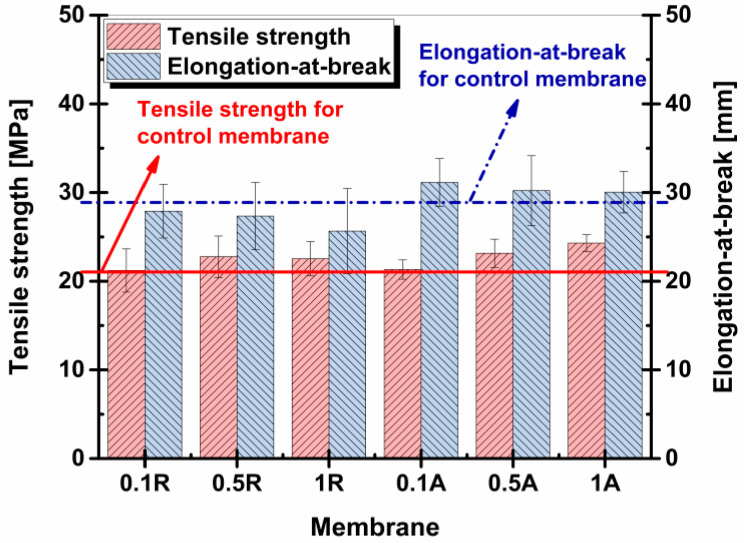
Tensile strengths and elongations-at-break of membranes with the addition of TiO_2_ rutile and anatase (Note: Table 19.61 ± 1.8 MPa and 31.53 ± 1.3 mm, respectively).

Figure 10 introduces the details regarding membrane elongation until specimen rupture. It can be observed that elongation-at-break values were inversely proportional to increases in nanoparticle concentration. 

In the case of the anatase allotrope, as shown in the interpretation of Raman spectroscopy results (Section 3.2), the disappearance of a vibration mode could mean that there was a strong interaction between this type of nanoparticle and the polymeric matrix. The higher elongation-at-break values are explained by the more significant presence of nanoparticles in the membrane structure as confirmed by EDX investigations (Table 2), as well as their satisfactory distribution in the membrane matrices (Figure 4). These factors could contribute to the creation of a matrix–filler bond, resulting in intensification in controlled dislocations of the polymeric chains without the appearance of defects leading to rapid breaking of samples. 

In the case of TiO_2_ rutile membranes, results showed a decrease elongation-at-break with a higher concentration of nanoparticles, because these nanoparticles limited the independent movement of the polymer chains without weakening them. This aspect could be explained by the absence of chemical interaction between nanoparticles and the polymeric matrix, as confirmed by Raman analysis, where the increases in peak intensity characteristic of rutile nanoparticles were observed in the membrane without any of the vibration modes of this structure being attenuated or eliminated. The lack of interaction with the matrix would not allow the transfer of forces from the matrix to the nanoparticles, so that the polymer chains would detach from the nanoparticles that became instigators of micro-cracks.

#### 3.5.2. Tensile Strength

The incorporation of TiO_2_ nanoparticles at a low concentration (0.1 wt.%) did not influence the nanocomposite membranes in terms of tensile strength, regardless of their crystalline structure (Figure 10). However, the increase in nanoparticle concentration in the PSf matrix resulted in improvement in the breaking resistance of nanocomposite membranes compared to the control membrane.

By increasing the content of TiO_2_ from 0.1 wt.% to 0.5 wt.%, the tensile strength increased from 21.2 MPa to 22.8 MPa for membranes modified with TiO_2_ rutile, and from 21.3 MPa to 23.2 MPa in the case of anatase. Zhu et al. [59] reported that this increment in tensile strength of nanocomposite membranes was attributed to the formation of an intercalated structure that limited the mobility of the polymer chains in the membrane. This intercalation could validate the lower values of elongation-at-break as well.

Increasing the concentration of nanoparticles to 1 wt.% produced different behaviors between rutile and anatase. The tensile strength of the 1 wt.% TiO_2_ anatase blended membrane increased to 24.3 MPa, 23.9% higher compared to the control membrane. This nanocomposite membrane obtained the largest value of tensile strength. In the case of the 1 wt.% TiO_2_ rutile membrane, the tensile strength value slightly decreased to 22.5 MPa.

Due to the better compatibility of the TiO_2_ anatase nanoparticles with the polymeric matrix, it appears that the crystalline structure of anatase resulted in the formation of membranes with better mechanical properties.

### 3.6. Compaction Factor

The compaction factor of the studied membranes provides details regarding the pressure resistance during the filtration of distilled water. This parameter should only be analyzed in the case of pure water filtration. Otherwise, if the water subjected to separation includes certain contaminants, it is not possible to discuss merely the compaction of the membranes, since other factors such as membrane fouling can affect this value.

A substantial degree of compaction implies a significant decrease in flux during filtration tests, as can be seen for the control membrane (Table 6). One of the reasons for flux decline can be directly related to the morphology of macrovoids, namely the difference in their thickness across the entire membrane cross-section. From the morphological analysis provided by cross-section SEM images of the control membrane (Figure 1), it can be observed that the volume of a single macrovoid in the control was relatively higher than those in nanocomposite membranes (Figure 2). This could lead to the weakening of mechanical properties of the control membrane, in particular the pressure resistance during filtration, because of the possible pore blockages caused by the narrowing of macrovoids. Hosseini and his colleagues (2018) [38] analyzed the degree of membrane compaction of 13 wt.% and 17 wt.% polyethersulfone with the addition of TiO_2_ nanoparticles (5, 7, 10 wt.%). They stated that the membranes with a superior degree of compaction were susceptible to irreversible physical compaction, which demonstrated the existence of defects as well as of enlarged macrovoids in the membrane structure, and that during filtration there was a compression of the active layer and macrovoids, producing a flux decrease.

It is also observed that the membranes modified with TiO_2_ rutile showed lower degrees of compaction than the control and TiO_2_ anatase membranes. This aspect can show a connection with the distribution of nanoparticles in the membrane cross-section observed via EDX maps. Although the presence of anatase TiO_2_ nanoparticles was higher in the membrane matrix (Table 2), their distribution was relatively uneven (Figure 3a–c) compared to the membranes with the addition of rutile TiO_2_ (Figure 3d–f). When discussing membrane compaction during filtration, it is noteworthy to study the uniformity of nanoparticles throughout the membrane structure. Incorporation of nanoparticles, as well as increasing their concentration in polymeric solutions, will produce membranes with higher mechanical strength and lower compaction factors, resulting in constant water flux during filtration.

The compaction factor may belong to the mechanical stresses in the elastic range of the material due to the relatively low operating pressures.

In addition, in the absence of mechanical tests, the compaction factor can represent a practical tool in investigating the influence of nanoparticles on membranes from a mechanical point of view.

### 3.7. Determination of Total Performance

**Figure 11:**
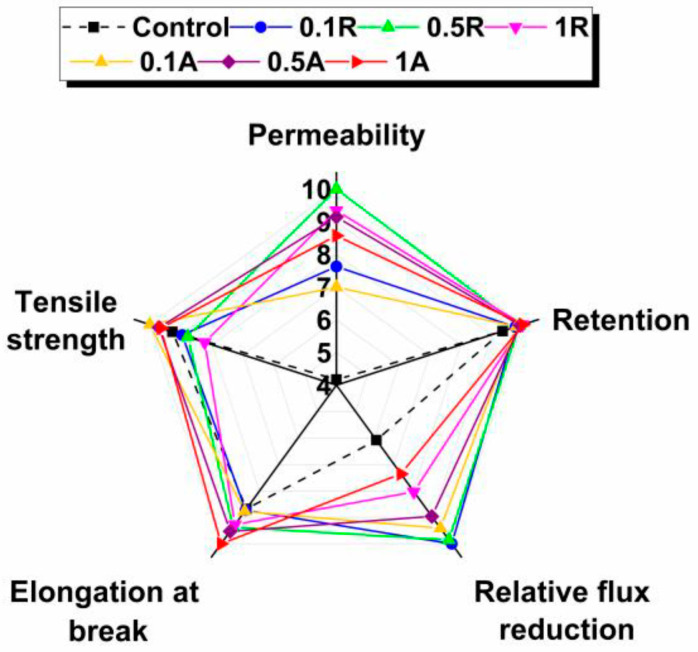
Performance of control membrane and membranes with nanoparticle additions of TiO_2_ rutile and anatase.

For qualitative analysis, the obtained values of each parameter were transformed into ratings from 0 to 10 and displayed in a radar chart, as can be seen in Figure 11. It can be observed that following the displaying of values, different irregular polygons were formed for each membrane, resulting in different areas of optimal performance. The control membrane had the most compact area, which means that most of its parameters had the lowest values. The nanocomposite membranes obtained higher performance areas, which visually confirms the effective influence of nanoparticles.

Because the differences between the two types of nanoparticles were not substantial, it is quite challenging to find the optimum membrane only from analyzing the graph. We considered there to be an ideal membrane with all the parameters marked with 10. Thus, the ideal performance area of this simulated membrane would comprise the combined area of the pentagon. By establishing the ratios between the real areas produced by the performances of the membranes and the ideal area, the values can be quantified in performance indices (PI), as shown in Table 7.

It can be noted that the best membrane was the one modified with 0.5 wt.% TiO_2_ rutile, the next being the 0.5 wt.% TiO_2_ anatase-blended membrane. The difference between the two membranes was negligible, the membrane with rutile recording an increase of only 2.8 %. However, it can be concluded that a concentration of 0.5 wt.% TiO_2_ nanoparticles showed the most satisfactory performances regardless of crystalline structure. Comparing the performance index of the control membrane with that of the best membrane from this study, an increase of 71.1% was achieved.

## 4. Conclusions

Based on the modification of membranes by the addition of titanium dioxide nanoparticles with rutile and anatase crystalline structures, the following conclusions can be highlighted.

Substantial changes were observed from the structural point of view. The most notable observations were the increase in pore number, decrease in pore size, and increase in porosity of the modified membranes. These characteristics were associated with the increased permeability of the nanocomposite membranes compared to the control membrane.

The higher mass percentage of anatase TiO_2_ nanoparticles identified by EDX analysis resulted in increased hydrophilicity, and the higher agglomeration tendency of anatase TiO_2_ nanoparticles caused an increase in surface roughness. These investigations were consistent with a higher stability of relative flux over time, but a greater degree of fouling for the anatase TiO_2_ membranes compared to the rutile TiO_2_ membranes.

The retention degree of Congo Red dye by the nanocomposite membranes was over 95%. Retention values were directly proportional to the mass percentage of nanoparticles incorporated. These results were confirmed by SEM surface micrographs.

Mechanical tests showed a positive impact of nanoparticles on membrane integrity. All nanocomposite membranes had superior mechanical properties to the pure membrane. Regarding nanoparticle crystalline structure, it was found that the membranes with the addition of TiO_2_ anatase obtained the highest tensile strengths and elongations-at-break.

From the calculated total performance for each membrane, it was found that the difference between crystalline forms of TiO_2_ nanoparticles was not noticeable, the membrane with 0.5 wt.% TiO_2_ rutile achieving a performance only 2.8% higher than the membrane with 0.5 wt.% TiO_2_ anatase.

We can conclude that the role of crystalline structure in obtaining a membrane with optimal properties cannot be considered significant, at least when discussing the crystalline structures of titanium dioxide. When compared with the control membrane, the optimal membrane was produced by blending 0.5 wt.% TiO_2_ rutile nanoparticles into the polymer solution.

Resembling the titan Atlas of Greek mythology, who was condemned to hold Gaia (Earth) with his endurance and power, we could say that even in this study, nanoparticles could play the role of sustaining polymer chains through the high energy of these nano-bodies. It is harmonious to remark that the material of interest is named after the Titans of Greek mythology. In this context, the nanoparticles deserve their name.

## Figures and Tables

**Table 1:** Concentrations of added nanomaterials in the polymeric solutions.

Membrane Code	NP Conc. [wt.%]	Crystalline Structure
Control	-	-
0.1R	0.1	rutile
0.1A		anatase
0.5R	0.5	rutile
0.5A		anatase
1R	1	rutile
1A		anatase

**Table 2:** Elemental analysis of titanium via EDX method on surface (**a**) and in cross-section (**b**) of control membrane and membranes with the addition of titanium dioxide nanoparticles.

Membrane	(a) Top-View	(b) Cross-Section
	Titanium [%]	Titanium [%]
Control	-	-
0.1R	0.04 ± 0.01	0.03 ± 0.02
0.5R	0.11 ± 0.05	0.35 ± 0.12
1R	0.52 ± 0.07	0.55 ± 0.14
0.1A	0.14 ± 0.04	0.16 ± 0.05
0.5A	0.20 ± 0.25	0.27 ± 0.11
1A	0.88 ± 0.06	0.43 ± 0.29

**Table 3:** Assignment of polysulfone characteristic Raman peaks with possible functional groups or specific molecular vibrations, collected from the literature [39,40,41,42,43,44].

Raman Peak [cm^−1^]	Functional Group/Molecular Vibrations
644	Stretching vibration of C-S aliphatic chain
	Vibration of asymmetric deformation of C-S-C bond
795	Out-of-plane asymmetric displacement vibrations of C-H bond in the benzene ring
	Aliphatic C-C chain deformation vibration
1080	Planar vibration of C-H bond in benzene ring
	Stretch vibration of aromatic C-S bond
	Symmetrical stretch vibration of SO_2_ bond
1114	Asymmetrical stretch vibration of C-O-C bond
	Asymmetrical stretch vibration of SO_2_ bond
1151	Benzene ring breathing vibration mode coupled with C-S and C-O linkage movements
	Symmetrical deformation vibration of C-O-C bond
1591	In-plane deformation vibration of benzene rings

**Table 4:** Water contact angle, porosity, and roughness of the studied membranes.

Membrane	Water Contact Angle [°]	Porosity, ε [%]	Roughness, S_a_ [nm]
Control	73.6 ± 3.6	45.3 ± 7.4	4.4 ± 0.5
0.1R	66.6 ± 2.4	46.7 ± 8.1	3.8 ± 0.2
0.5R	61.6 ± 3.3	52.1 ± 6.5	3.8 ± 0.8
1R	60.5 ± 2.3	57.4 ± 6.6	5.7 ± 1.7
0.1A	64.8 ± 2.2	44.5 ± 9.1	4.1 ± 0.6
0.5A	61.4 ± 1.7	49.2 ± 8.7	4.2 ± 1.1
1A	57.7 ± 1.1	52.2 ± 12.2	7.7 ± 1.9

**Table 5:** Relative flux reduction of the studied membranes.

Membrane	RFR [%]
Control	54.3 ± 1.9
0.1R	24.8 ± 2.4
0.5R	25.9 ± 2.6
1R	39.6 ± 2.4
0.1A	29.3 ± 2.1
0.5A	32.6 ± 2.6
1A	44.7 ± 2.1

**Table 6:** Compaction degree during filtration for the studied membranes.

Membrane	Compaction Factor [%]	Standard Deviation
Control	32.5	±2.9
0.1R	17.3	±2.9
0.5R	11.6	±0.4
1R	8.5	±1.1
0.1A	23.1	±1.9
0.5A	15.9	±0.8
1A	13.1	±0.8

**Table 7:** Analysis of total membrane performance results regarding the incorporation of titanium dioxide nanoparticles.

Membrane	PI
Control	0.536
0.1R	0.812
0.5R	0.917
1R	0.803
0.1A	0.802
0.5A	0.892
1A	0.824

## Data Availability

The datasets generated for this study are available upon request to the corresponding author.

## Supplementary Materials

The following are available online at https://www.mdpi.com/article/10.3390/membranes11110841/s1, Figure S1: XRD patterns for the commercial nanoparticles of this study: (A) nanoparticles composed of anatase and rutile mixture and (B) rutile nanoparticles, Figure S2: Raman spectra of the two commercial titanium dioxide nanoparticles with rutile and anatase crystalline phases.
