# Supplementary material for: New Understanding of the Difference in Filtration Performance between Anatase and Rutile TiO2 Nanoparticles through Blending into Ultrafiltration PSF Membranes
Source: Membranes (Basel). 2021 Oct 29;11(11):841. doi: 10.3390/membranes11110841 (PMC8625676; doi:10.3390/membranes11110841)
Supplement: Supplementary file 1 [file membranes-11-00841-s001.zip › membranes-1388194-SI.pdf]

Supplementary materials

New Understanding of the Difference in Filtration Performance between Anatase and Rutile TiO_2 Nanoparticles through Blending into Ultrafiltration PSF Membranes

Iulian-Gabriel Birsan [1], Stefan Catalin Pintilie [1,*], Laurentia Geanina Pintilie [2], Andreea Liliana Lazar [2], Adrian Circiumaru [1] and Stefan Balta [2,*]

1 Department of Applied Sciences, Cross-border Faculty, Dunarea de Jos University of Galati, 111th Domneasca Street, 800201 Galati, Romania; iulian.birsan@ugal.ro (I.-G.B.); adrian.circiumaru@ugal.ro (A.C.)

2 Department of Materials and Environmental Engineering, Faculty of Engineering, Dunarea de Jos University of Galati, 111th Domneasca Street, 800201 Galati, Romania; geanina.pintilie@ugal.ro or tironglaurentia@yahoo.com ((L.G.P.); andreea.lazar@ugal.ro (A.L.L.)

* Correspondence: stefan.pintilie@ugal.ro or pintiliescatalin@gmail.com ((S.C.P.); stefan.balta@ugal.ro (S.B.)

1. Methods of Characterization for the Studied Nanopowders

S1. The XRD method was used to determine the phase composition of the commercial TiO_2 nanopowders. Diffraction was obtained with a DRON-3M diffractometer (Saint-Petersburg, Russia), equipped with a molybdenum anticathode (Mo-Kα1).

S2. The preconfigured Raman spectrometry system (StellarNet, Tampa, USA), composed of the Raman-HR-TEC-785 spectrometer connected to the Ramulaser 785 nm laser, was used to analyze the structure of the commercial nanopowders.

2. Results

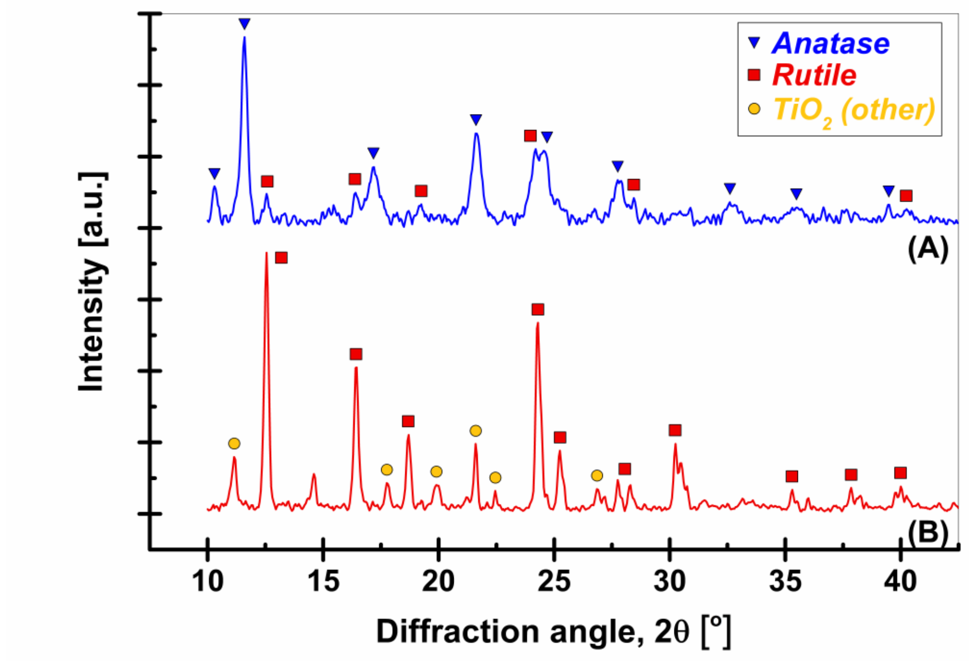

Figure S1. XRD patterns for the commercial nanoparticles of this study: (A) nanoparticles composed of anatase and rutile mixture and (B) rutile nanoparticles

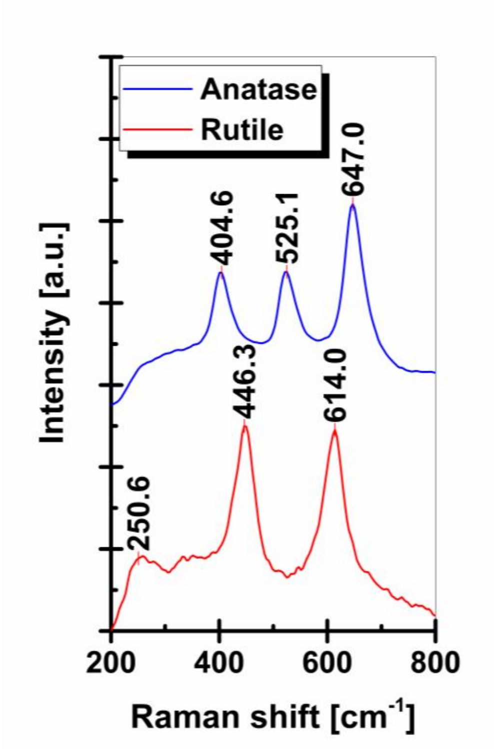

Figure S2. Raman spectra of the two commercial titanium dioxide nanoparticles with rutile and anatase crystalline phases
